# Application of Allogeneic Human Acellular Dermal Matrix Reduces the Incidence of Fistula in Hypospadias Repair

**DOI:** 10.3389/fped.2022.774973

**Published:** 2022-03-09

**Authors:** Shijian Wu, Chenglong Ye, Huai Yang, Bote Chen, Haibo Nie, Shaowei Li

**Affiliations:** ^1^Department of Urology, General Hospital of Southern Theatre Command, Guangzhou, China; ^2^Graduate School, Guangzhou University of Chinese Medicine, Guangzhou, China; ^3^Department of Urology, Guangdong Second Provincial General Hospital, Guangzhou, China

**Keywords:** human acellular dermal matrix, HADM, hypospadias, tubularized incised plate, TIP, urethrocutaneous fistula

## Abstract

**Background:**

Urethrocutaneous fistula is one of the most common complications arising from hypospadias surgery. The purpose of our study was to investigate the effectiveness and safety of allogeneic human acellular dermal matrix (HADM) application as a means of preventing the occurrence of urethrocutaneous fistula following hypospadias surgery.

**Methods:**

This is a non-randomized study of 219 cases (out of 270 patients with hypospadias) which satisfied inclusion and exclusion criteria. These patients were divided into two groups: 101 HADM patients, and 118 control patients (who did not receive HADM). In the control group, 77 boys were treated by single-stage urethroplasty (TIP) and 41 underwent staged urethroplasty (Thiersch-Duplay). In the HADM group, 59 boys underwent the TIP and 42 underwent the Thiersch-Duplay. In the postoperative period, we recorded the incidence of infection, urethrocutaneous fistula, and urethral stricture complications in these two groups of patients. The effectiveness and safety of HADM in preventing urethrocutaneous fistula following hypospadias surgery were evaluated according to these indicators.

**Results:**

In the control group, following the operation there were 16 cases of infection, 38 cases of urethrocutaneous fistula after extubating, and 5 cases of urethral stricture. In the HADM group, there were 19 cases of postoperative infection, 12 cases of urethrocutaneous fistula after extubating, and 5 children with urethral stricture. In comparing the two groups, it was found that the postoperative infection rate (13.6 vs. 18.8%) and the incidence of urethral stricture (4.2 vs. 5.0%) were not statistically significant (*P* > 0.05), while the postoperative urethrocutaneous fistula rate (32.2 vs. 11.9%) was statistically significant (*P* < 0.001).

**Conclusion:**

It is found that HADM application can significantly reduce the incidence of urethrocutaneous fistula complications, without increasing the risk of infection and urethral stricture.

## Introduction

Hypospadias is a commonly occurring congenital malformation of the genitourinary system in males. Complications such as infection, urethrocutaneous fistula, urethral stricture, and urethral diverticulum are easy to occur after surgery. The most commonly reported serious complications after hypospadias surgery are urethral stricture and urethrocutaneous fistula, both of which require surgical treatment again (1). Hypospadias can be classified as distal, midpenile, and proximal according to the preoperative meatal position. Proximal and midpenile hypospadias have a higher incidence of postoperative urethrocutaneous fistula than distal hypospadias.

To reduce the incidence of fistula, covering the neourethra with adjacent tissues such as fascia, dartos, and penile flap is most reliable. Recently, medics have tried to repair hypospadias with buccal mucosa (2, 3). Springer et al. (4) shared their experience of repairing urethrocutaneous fistula with an acellular collagen matrix graft and considered it a safe and effective adjunct to prevent further recurrence of fistula.

HADM is a natural acellular dermal substitute of allogenic dermis after acellular treatment. HADM is both cell-free and major histocompatibility complex (MHC) -free; it does not trigger immune rejection, and it has good histocompatibility. To ensure the safety and efficacy of HADM in clinical applications, investigators have evaluated its possible host-graft rejection and fibrotic reactions in animal experiments (5, 6). To date, HADM has been successfully used in the repair of urethral stricture, with positive clinical effects (7).

This study aims to assess the effectiveness and safety of HADM in hypospadias repair.

## Methods

### Patient Information

#### Patient Selection

**Figure 1:**
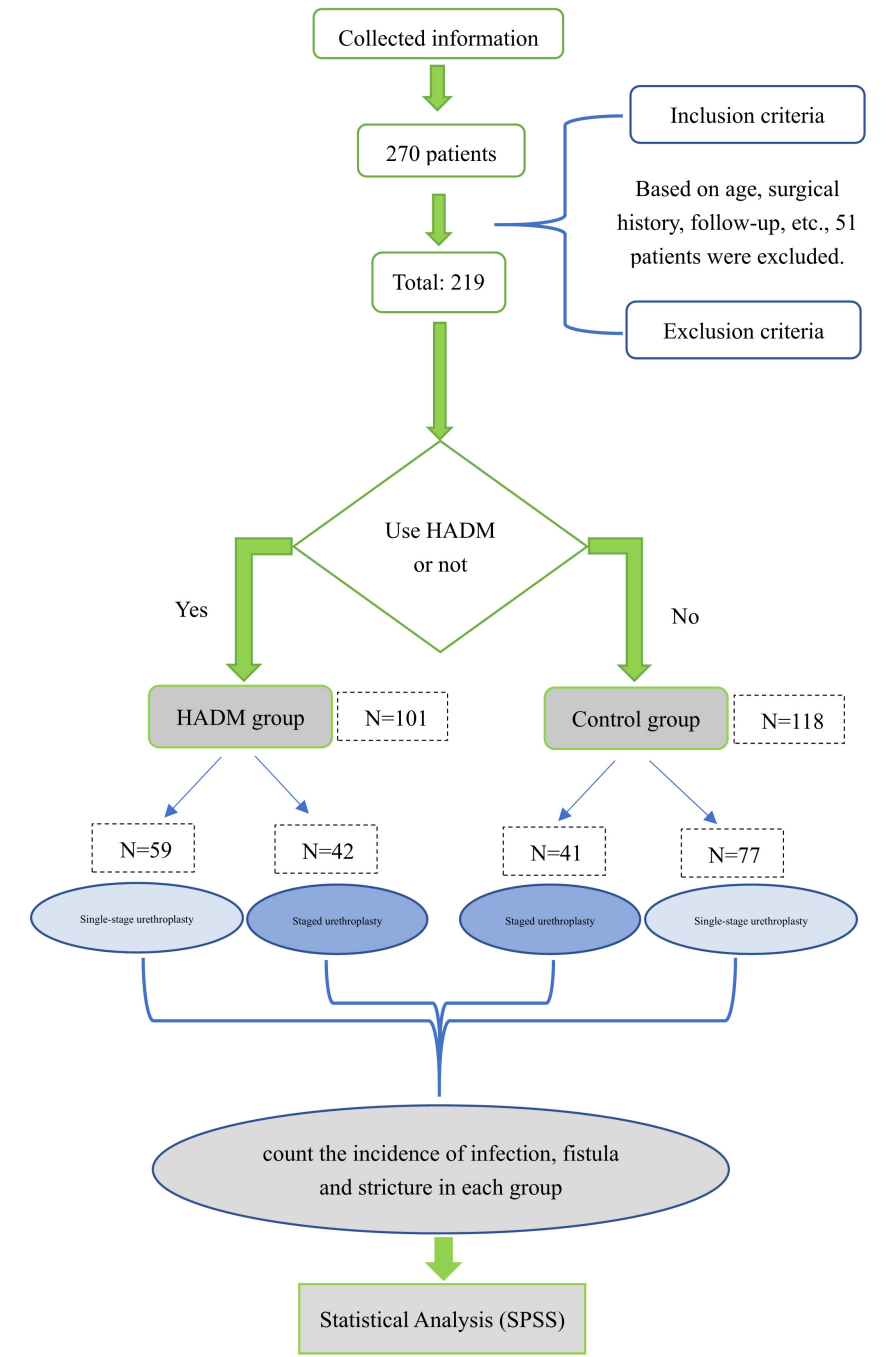
Flow chart.

We screened 219 patients with hypospadias (control group: 118 cases, HADM group: 101 cases) based on the inclusion and exclusion criteria (from January 2017 to July 2020). All patients were treated by the same surgical team at our institution: of the 101 patients in the HADM group, 59 patients underwent single-stage urethroplasty and 42 patients underwent staged urethroplasty. Of the 118 patients in the control group, 77 patients underwent single-stage urethroplasty, and 41 patients underwent staged urethroplasty (Figure 1). The complications including urethrocutaneous fistula, urethral stricture and infection were recorded. Urethrocutaneous fistula was taken as the primary outcome index, and urethral stricture and infection as the secondary outcome indexes.

#### Inclusion Criteria

We selected patients who met the following criteria: (i) patients who underwent single-stage urethroplasty (TIP) or staged urethroplasty (Thiersch-Duplay); (ii) patients aged 19–60 months; (iii) patients who had been approached following full communication with the surgeon, and whose family voluntarily signed an informed consent form; (iv) patients who had undergone completion of full treatment and follow-up for 12 months or more; and, (v) patients who had been operated on by the same surgical team.

#### Exclusion Criteria

Patients belonging to the following categories were excluded from this study: (i) patients with hypospadias who have undergone urethroplasty in other hospitals; (ii) those who had undergone other procedures such as orchidopexy with urethroplasty; (iii) those with an incomplete follow-up; and, (iv) those with distal hypospadias.

### Source and Preparation of HADM

The HADM was derived from young people's skin, prepared by Jayyalife Biological Technology (Beijing, China) and stored at 2–8 degrees Celsius. Its use was approved by the China Food and Drug Administration. The surface morphology of HADM was analyzed using a scanning electron microscope (Quanta 450FEG, FEI).

### Surgical Procedure

All patients underwent single-stage urethroplasty (TIP) or staged urethroplasty (Thiersch-Duplay). TIP was performed for hypospadias with flat glans and straight penis. For proximal hypospadias with severe penile curvature, staged urethroplasty was performed: penile curvature correction as a one-stage procedure, Thiersch-Duplay urethroplasty as a two-stage repair.

#### TIP

**Figure 2:**
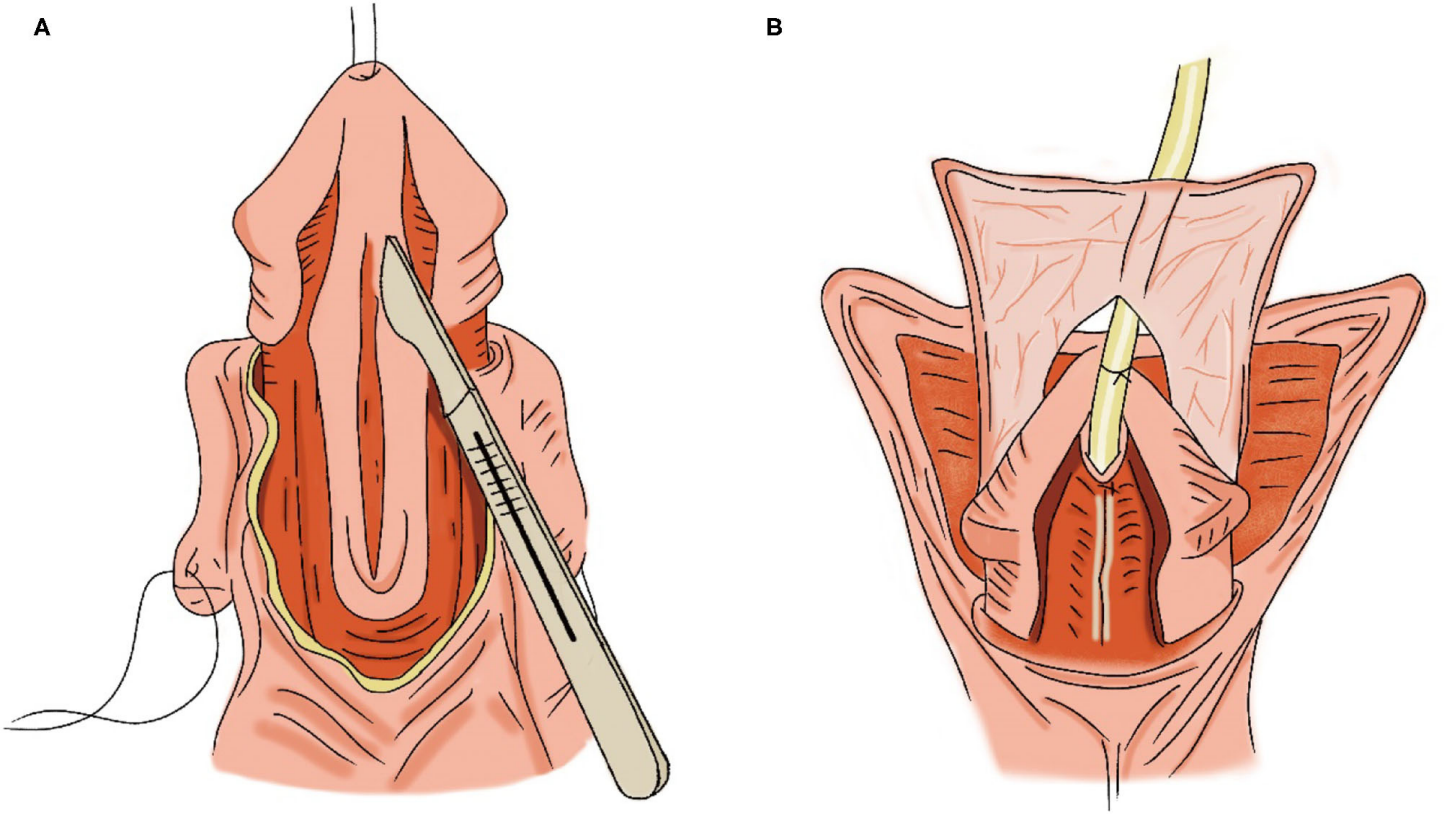
TIP [modified from Snodgrass et al. (8). p. 95]. **(A)** Midline incision of the urethral plate. **(B)** Pedicled tissue was used to cover the neourethra.

Surgery was done under general anesthesia. A traction suture was placed into the glans and a U-shaped skin incision was made along the edges of the urethral plate. The penile was completely degloved and then an artificial erection was performed to confirm the absence of ventral curvature. A midline incision was made to widen the urethral plate along its length to 1.0–1.2 cm (Figure 2A). A silicone catheter (F8) was inserted through the hypospadic meatus as a stent, and the urethral plate was then tubularized over the stent. Pedicled tissue was dissected from the dorsal penile skin and used to cover the neourethra (Figure 2B). Finally, closure of the ventral penile skin was finished in the midline (8).

#### Thiersch-Duplay

**Figure 3:**
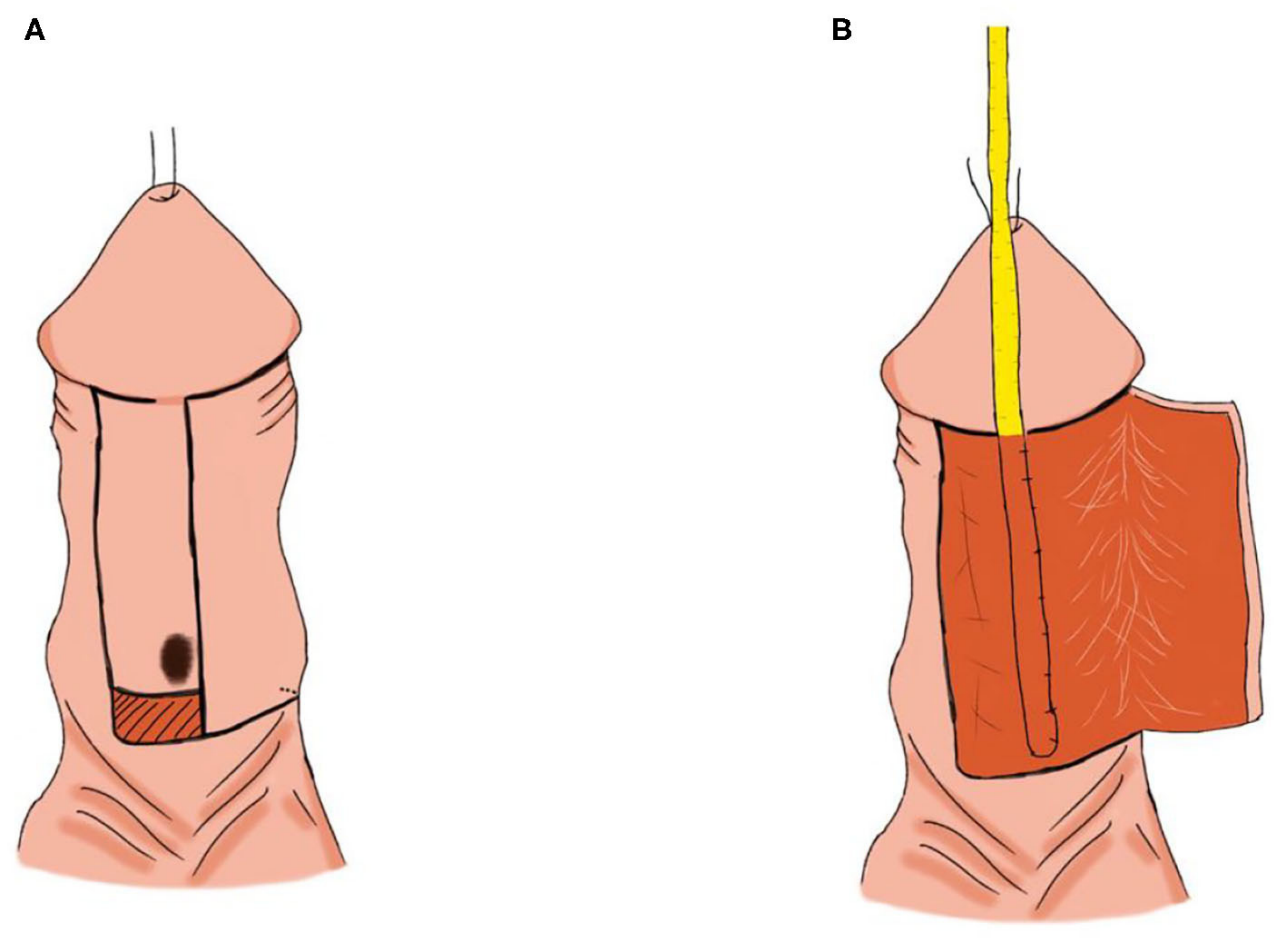
Duplay. **(A)** U-shaped skin incision. **(B)** The flap was tubularized over the stent.

Surgery was performed under general anesthesia. A traction suture was placed into the glans and a U-shaped skin incision was made in the ventral penile skin around the hypospadic meatus. One side of the incision was close to the midline, and the other side was far from the midline, with a width of about 1.0–1.2 cm. Three more transverse incisions were made as shown in Figure 3A. Then the ventral flap was dissected, a silicone catheter (F8) was inserted through the hypospadic meatus as a stent, and the flap was then tubularized over the stent (Figure 3B). The dartos was dissected and covered over the neourethra. Finally, the skin incision was closed.

**Figure 4:**
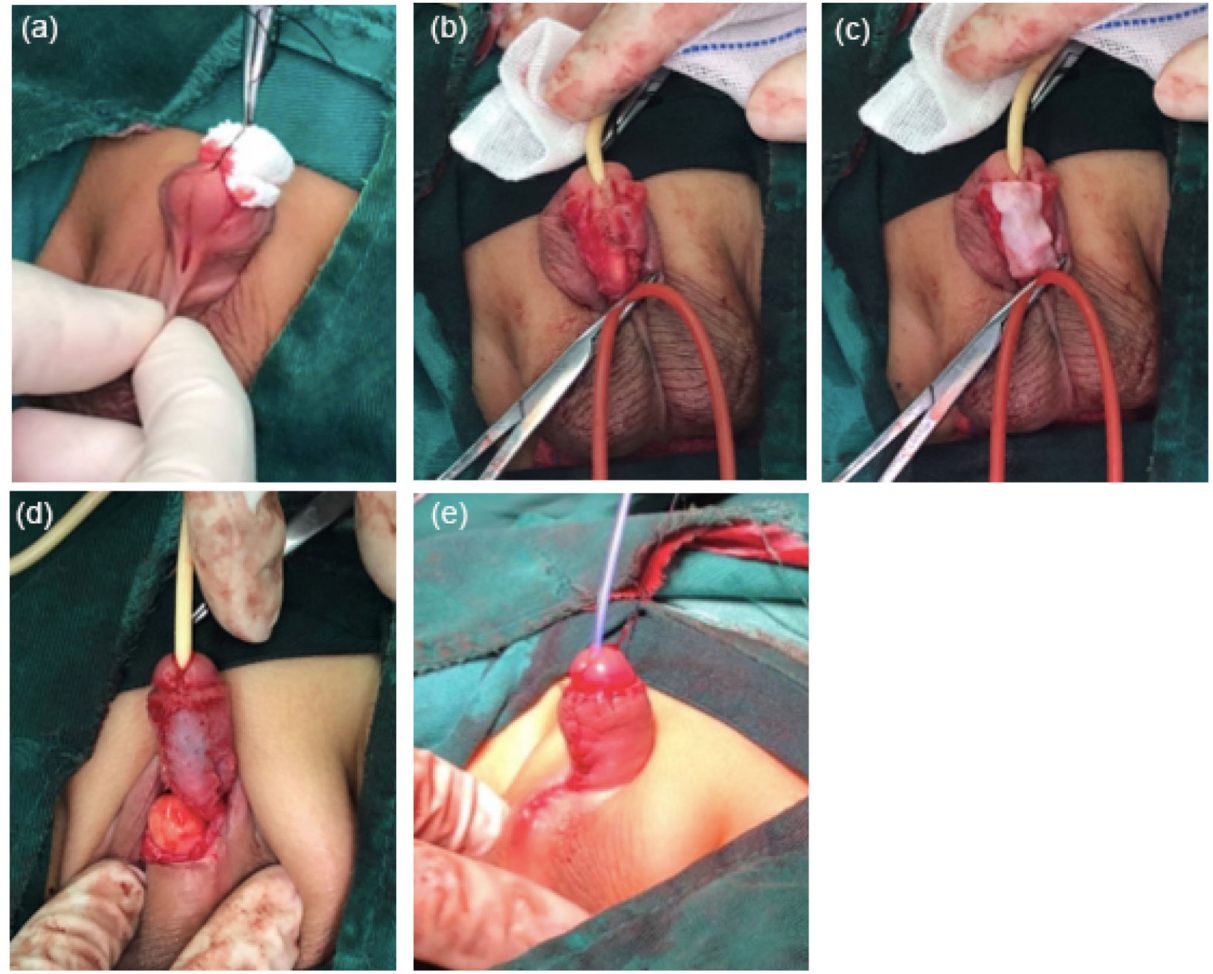
Procedures to supplement HADM. **(a)** 2 years and 3 months of children with midpenile hypospadias. **(b)** The urethral plate was tubularized over the stent. **(c)** HADM covered the neourethra. **(d)** Polyglycolic acid sutures sutured and fixed the HADM. **(e)** Postoperative appearance.

Patients from the control group received TIP or Thiersch-Duplay. The HADM group performed the same procedure but added a waterproof layer between the dartos and the neourethra (Figure 4).

The same surgical team operated on all patients, and preoperative and postoperative care was identical. All patients had their wound dressings removed on the fifth postoperative day and the urinary catheter was removed 14 days after surgery. Prophylactic antibiotic therapy was performed with cefuroxime sodium or piperacillin-tazobactam for all patients prior to catheter removal. After the removal of the catheter, the patients underwent a urodynamics inspection.

### Follow-Up

All patients were followed up for at least 12 months, with a mean follow-up of 18 months. Clinical assessment was performed for the first spontaneous urination after catheter removal. The patients were followed up at 1, 3, 6, 9, and 12 months intervals and half-yearly thereafter. Incidences of infection, urethrocutaneous fistula, and urethral stricture were assessed by two surgeons during each outpatient visit.

### Statistical Analysis

We evaluated these patients by age, length of the neourethra, and complications (such as infection, urethrocutaneous fistula and urethral stricture). The primary outcome was urethrocutaneous fistula. A sample size of 44 single-stage urethroplasty patients in the HADM group was estimated for this prospective study, which was calculated based on α = 0.05 and β = 0.20, Alternative Hypothesis (H1): One-sided (H1:D1>0), and a predicted P1 = 0.90 (according to the preliminary results of this study). The sample size of staged urethroplasty in the HADM group was estimated to be 41 patients with P1 = 0.85. The study eventually yielded a total sample size of 101. The power was calculated by Power Analysis & Sample Size (PASS) software. Alpha = 0.05, Alternative Hypothesis (H1): One-sided (H1:D1>0), Test Type: Z Test (Pooled), Power = 0.8682 (single-stage urethroplasty), Power = 0.8375 (staged urethroplasty). The data were analyzed using the Statistical Product and Service Solutions (SPSS) software package (Windows version 24) (SPSS Inc., IL, USA). Differences between the groups were compared using the Chi-square test, for which *P* < 0.05 was considered to be statistically significant.

## Results

### Structure of Human Acellular Dermal Matrix

**Figure 5:**
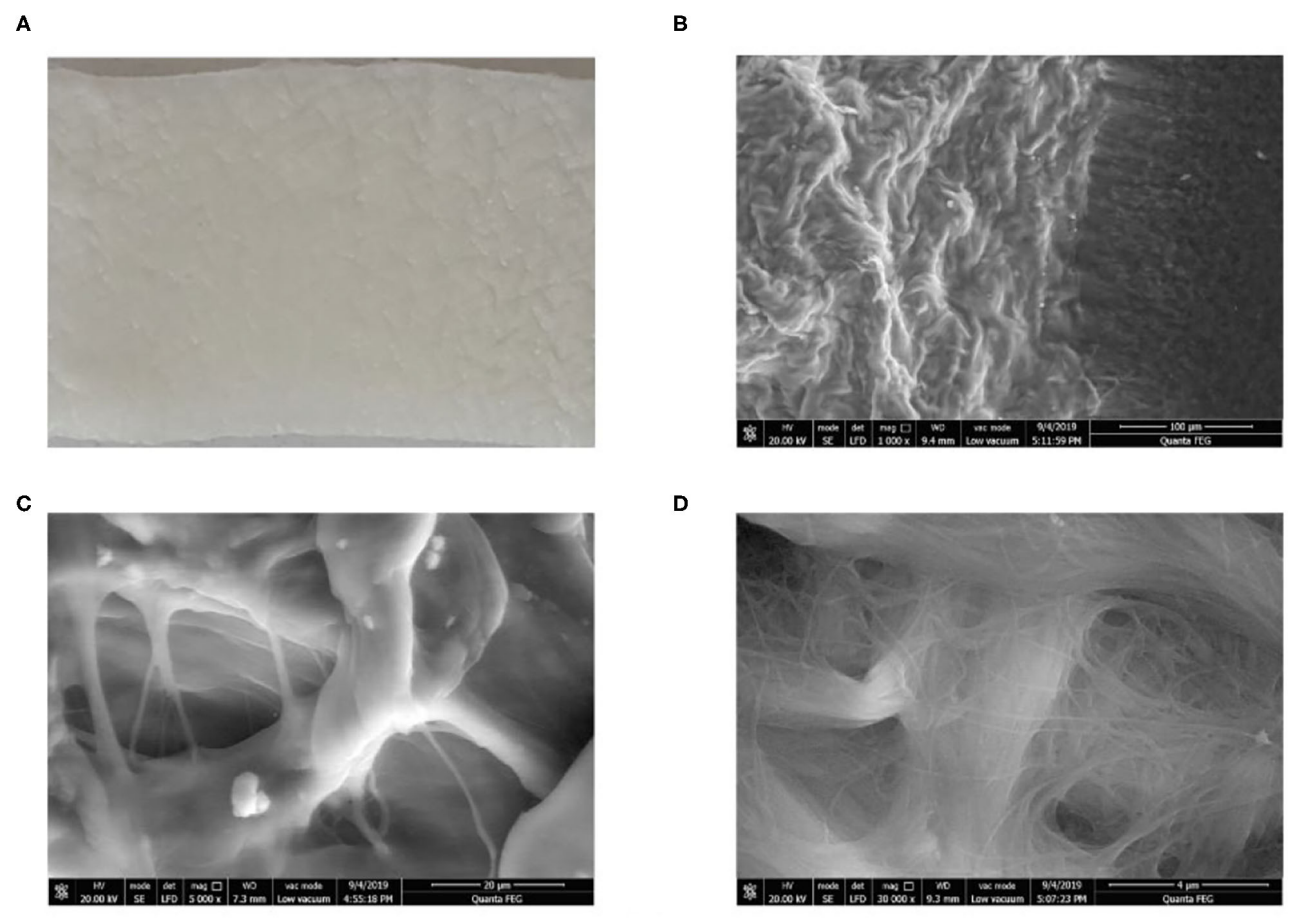
Appearance and structure of HADM. **(A)** Appearance of HADM. **(B–D)** Scanning electron microscopy showed HADM magnifications of 1,000, 5,000, and 30,000.

The appearance of HADM is shown in Figure 5A. The HADM exhibited a porous stereoscopic structure. Scanning electron microscopy results are shown in Figures 5B–D.

### Comparisons of Postoperative Complications Among the Groups

In each case, the catheter was removed 2 weeks after surgery. The occurrence of three complications is shown in Tables 1–3. It was found that HADM application did not significantly increase the risk of postoperative infections and urethral stricture (*P* > 0.05), but significantly reduced the incidence of urethrocutaneous fistula (*P* < 0.05).

**Table 1:** Comparisons of postoperative complications among the groups.

	**HADM group**	**Control group**	***P***
Patients	101	118	
Age, months	37.09 ± 10.56	36.90 ± 8.49	0.884
Length of the neourethra, cm	2.36 ± 0.62	2.24 ± 0.62	0.154
Infection	19 (18.8%)	16 (13.6%)	0.29
Urethral stricture	5 (5.0%)	5 (4.2%)	1.000
Urethrocutaneous fistula	12 (11.9%)	38 (32.2%)	<0.001

**Table 2:** Comparisons of complications after single-stage urethroplasty.

	**Single-stage urethroplasty of the HADM group**	**Single-stage urethroplasty of the control group**	***P***
Patients	59	77	
Age, months	33.85 ± 9.79	34.56 ± 7.62	0.635
Length of the neourethra, cm	1.87 ± 0.25	1.82 ± 0.26	0.245
Infection	11 (18.6%)	10 (13.0%)	0.366
Urethral stricture	3 (5.1%)	3 (3.9%)	1.000
Urethrocutaneous fistula	6 (10.2%)	22 (28.6%)	0.009

**Table 3:** Comparisons of complications after staged urethroplasty.

	**Staged urethroplasty of the HADM group**	**Staged urethroplasty of the control group**	***P***
Patients	42	41	
Age, months	41.64 ± 10.01	41.29 ± 8.37	0.863
Length of the neourethra, cm	3.05 ± 0.20	3.03 ± 0.20	0.642
Infection	8 (19.0%)	6 (14.6%)	0.591
Urethral stricture	2 (4.8%)	2 (4.9%)	1.000
Urethrocutaneous fistula	6 (14.3%)	16 (39.0%)	0.011

### Safety of HADM for Hypospadias

All complications recorded in this study occurred within 1 year postoperatively, and no further complications were diagnosed after the 12-month follow-up visit. No evidence of graft-host rejection was found in the HADM group at the 12-month follow-up stage.

## Discussion

Hypospadias is a common congenital anomaly that occurs in ~1/250–1/300 live births (9). Over 300 techniques for repairing hypospadias have been developed and described (10). Most of these techniques are constantly being revised and modified in order to achieve ideal results, as the rate of postoperative complications remains high. To reduce the incidence of urethrocutaneous fistula, scholars worldwide have been developing and improving surgical technologies and styles, such as individual procedures during perioperative treatment (11–13). It is reported that the preoperative application of HCG and dihydrotestosterone ointment can improve the appearance of the penis and reduce the incidence of complications (14, 15). Changes to the surgical approach constitute another route for reducing the incidence of urethrocutaneous fistula. Commonly used surgical procedures for hypospadias include Duckett, Island Flap urethroplasty, Duplay and Onlay (16). In 1994, Snodgrass et al. (17) pioneered TIP urethroplasty, which is not only simple to perform, but has a higher operation success rate and guarantees improved appearance. For treatment of certain severe hypospadias, medics have developed penile curvature correction followed by two-stage urethroplasty; this too has yielded promising results (18). During the operation, the specific procedure is an important aspect of reducing the risk of postoperative fistula. Covering the neourethra with multiple layers during the operation has been reported to be a particularly effective method. Commonly used materials include the surrounding sarcoid or dartos fascia, pedicled skinned foreskin or scrotal flaps, and even testicular sheath flaps (19–21). We also used the tunica vaginalis spongiosum and dartos as covering layers, as Arshadi et al. (22) did. Given the high incidence of urethrocutaneous fistula in proximal hypospadias, we use as much of this healthy tissue as possible to cover the neourethra while ensuring a tension-free urethroplasty. Ghanem et al. (23) reported that they used the dorsal dartos flap to cover the neourethra in proximal TIP urethroplasty with satisfactory results. Chatterjee et al. (24) reported 29 cases of hypospadias with testicular sheath covering the neourethra, and no incidences of urethrocutaneous fistula occurring after surgery. Zhang et al. (25) reported on 18 cases of a scrotal-septal fasciocutaneous flap being used to cover the surface of anastomotic urethra, all achieving good results. These healthy tissues of autologous origin, especially the tunica vaginalis spongiosum, are the ideal replacement for the urethra. However, some patients lack these tissues. In most cases of midpenile hypospadias, we can see the ruptured cavernous body of the urethra. After suturing the neourethra and covering the HADM, the spongioplasty was performed. In proximal hypospadias, the ruptured cavernous body of the urethra is generally poorly developed, and we cannot perform the spongioplasty. In order to reduce the risk of fistula, we need a new replacement to cover the neourethra. Lin et al. (26) used bovine skin-derived acellular dermal matrix as coverage in proximal hypospadias repair; their results showed a significantly reduced risk of fistula formation. According to the results of a randomized controlled trial, the use of AlloDerm (27), a human-derived freeze-dried acellular dermis, for hypospadias and fistula repair achieved good results. In the present study, we used HADM as an intermediate waterproof layer during the operation to cover the neourethra in order to reduce the risk of postoperative urethrocutaneous fistula.

HADM is an allogeneic skin material. During HADM manufacture, tissue engineering technology is used to remove all cells in the epithelium and dermis to avoid rejection during implantation, while retaining type IV collagen with its biochemical and structural effects (28). The three-dimensional structure is composed of low antigen substances such as protein, elastin, and proteoglycan, and can be used as a good scaffold for the growth of epithelial cells, fibroblasts, and new blood vessels after transplantation (29). The first HADM preparation method was devised by Livesey et al. (30). In the same year, Wainwright (31) applied it to patients who had suffered burns. In their study, a total of 67 burn patients received acellular dermal transplantation, and 14 days later were examined and compared against an autologous transplantation control group. Histology studies have shown that fibroblast infiltration, neovascularization, and epithelialization can be performed without rejection. Currently, these methods are widely used in burns and plastic surgery, stomatology, otolaryngology-head and neck surgical treatment, neurosurgery, and general surgery (32) to replace missing mucous membrane or skin tissue, with positive clinical results.

Recognized benefits of HADM include its non-antigenicity, three-dimensional structure, and rapid vascularization. In hypospadias repair, HADM is used to cover the suture of the neourethra to make its own tissue grow and reduce the risk of urethrocutaneous fistula. As a graft, the safety of HADM should be considered. So far, we have found no granuloma or granulomatous inflammation in these patients. Analysis of the data has revealed that covering the neourethra with HADM significantly reduces the incidence of urethrocutaneous fistula without increasing the probability of infection or urethral stricture. Application of the pedicled genital flap or testicular sheath with the epidermis to cover the neourethra is a difficult procedure that requires highly normal local tissue conditions. By comparison, the use of HADM to cover the neourethra is a simple operation that has little impact on local tissues, requires a short operation time and leads to few complications, especially for patients with inadequate penile skin.

One limitation of this study is the relatively small number of cases, with only 59 TIP and 42 Duplay cases. Another limitation of this study is the duration of follow-ups. Spinoit et al. reported that 47.4% of the post-operative complications of hypospadias occur in the first year of follow-up (33). In our opinion, future follow-ups can help evaluate the long-term outcomes of our method, such as whether it can reduce the incidence of long-term complications. Our team is still following up with these patients. Long-term follow-up of these children will be conducted, and further follow-up results will be reported in the future. Therefore, more high-quality, multicenter randomized trials are warranted.

## Conclusion

This study describes the application of HADM as a covering for the neourethra during hypospadias repair. The use of HADM reduced the incidence of urethrocutaneous fistula, without increasing the risk of infection and urethral stricture. This approach could be applied in clinical practice as an ideal option for the treatment of hypospadias.

## Data Availability Statement

The raw data supporting the conclusions of this article will be made available by the authors, without undue reservation.

## Ethics Statement

The studies involving human participants were reviewed and approved by Ethics Committee of the General Hospital of Southern Theatre Command. Written informed consent to participate in this study was provided by the participants' legal guardian/next of kin.

## Author Contributions

The data were collected by SW. The data were analyzed by BC, HN, and SL. Operations were performed by SW, HY, and CY. SW and CY wrote the manuscript and prepared the figures. HY edited the manuscript. All authors contributed to the article and approved the submitted version.

## Funding

This work was supported by Science and Technology Projects of Guangzhou (201904010317).

## Conflict of Interest

All authors have completed the ICMJE uniform disclosure form.

## Publisher's Note

All claims expressed in this article are solely those of the authors and do not necessarily represent those of their affiliated organizations, or those of the publisher, the editors and the reviewers. Any product that may be evaluated in this article, or claim that may be made by its manufacturer, is not guaranteed or endorsed by the publisher.
